# The Janus-faced Nature of miR-22 in Hematopoiesis: Is It an Oncogenic Tumor Suppressor or Rather a Tumor-Suppressive Oncogene?

**DOI:** 10.1371/journal.pgen.1006505

**Published:** 2017-01-12

**Authors:** Alexander Arthur Wurm, Daniel G. Tenen, Gerhard Behre

**Affiliations:** 1 Division of Hematology and Oncology, Leipzig University Hospital, Leipzig, Germany; 2 Cancer Science Institute, National University of Singapore, Singapore; 3 Harvard Stem Cell Institute, Harvard Medical School, Boston, Massachusetts, United States of America; Cincinnati Children's Hospital Medical Center, UNITED STATES

Hematopoiesis is a complex, multistep process originating from hematopoietic stem cells and structured into different lineages. Cell fate decision is well organized and strictly regulated by extrinsic and intrinsic molecules (such as cytokines and transcription factors) whose deregulation is connected to leukemic transformation. In recent years, the importance of microRNAs in controlling hematopoiesis has been discussed [1]. Since their discovery in 1993 [2], microRNAs have been intensively studied in multidisciplinary research fields and have even attracted the attention of the pharmaceutical industry as potential therapeutic targets in various diseases. The first drugs based on microRNA inhibition have already been approved [3]. Of note, increasing numbers of scientific publications focus on their role in oncology. It has been shown that microRNAs have great impact on cancer initiation or prevention, and several candidates were described to act as either tumor suppressors or oncogenes. While certain microRNAs can act as either tumor suppressors or oncogenes in different tissues, the observation of contradictory functions of a single microRNA in the same tissue and even the same cell type is rare and unusual. Looking at the myeloid lineage in the hematopoietic system, miR-181a is such a candidate: while Hickey et al. postulated its tumor-suppressive function in acute myeloid leukemia (AML) [4], several other groups revealed the oncogenic potential of miR-181a in the myeloid background [5, 6].

And what about miR-22 in hematopoiesis? In 2013, Song et al. demonstrated that miR-22 expression is up-regulated in myelodysplastic syndrome (MDS) and in AML [7]. They disclosed its oncogenic potential using retroviral and transgenic mouse models that developed hematological malignancies (including myeloid leukemia) and showed that knockdown of miR-22 blocked proliferation in leukemic cells. Given its role as an oncogenic microRNA, additional studies would have been expected to explore the therapeutic potential in blocking miR-22 in MDS or in AML. Surprisingly, at the beginning of 2016, Jiang et al. observed a different function of miR-22 in myeloid cells [8]: they demonstrated its tumor-suppressive potential in various cell culture and in vivo systems and found lower expression of miR-22 in AML compared to healthy controls. Is it possible that miR-22 has two faces in one cell lineage?

In September 2016’s issue of *PLOS Genetics*, Shen et al. provided further insights into the complexity of miR-22 function during myelopoiesis and with respect to myeloid leukemia [9]. The authors demonstrated that miR-22 is up-regulated during monocytic differentiation in various cell culture systems, including differentiation of primary human hematopoietic stem and progenitor cells (HSPCs). Furthermore, they revealed that transcription factor PU.1 is the regulator of miR-22 during this process and underlined the importance of miR-22 for monocytic differentiation by gain- and loss-of-function experiments. Interestingly, miR-22 targets MECOM, a transcription factor that is involved in hematopoietic stem cell renewal [10]. The repression of MECOM in turn leads to increased c-Jun levels, a protein that interacts with PU.1 to promote monocytic differentiation [11]. Consistent with previously published data by Jiang et al. [8], the authors found decreased miR-22 levels in AML and proposed enforced expression of miR-22 as a potential therapeutic approach for AML patients. In conclusion, Shen et al. clearly demonstrated the importance of miR-22 for monocytic differentiation and its tumor-suppressor potential in myeloid cells.

It is difficult to combine all previous findings of miR-22 in hematopoiesis. While the first report gave strong evidence of a classical oncogenic function, recent studies support the opposite view. Is there any rationale that miR-22 can be both a tumor suppressor and an oncogene in the same cell type? Song et al. found increased miR-22 levels in AML [7], while both Jiang et al. and Shen et al. reported the opposite [8, 9]. AML is a heterogeneous disease with huge biological differences between different subtypes [12]. Gene expression correlations between AML and non-AML cells are therefore somehow difficult to interpret. Additionally, significant conclusions are sometimes dependent on the quality and number of the appropriate controls. But nevertheless, while the observations by Song et al. are mainly based on experiments using transgenic mice with a nonleukemic background [7], Shen et al. focused exclusively on human cells [9]. Furthermore, the specific function of a single microRNA is always dependent on the expression of potential target mRNAs, on the accessibility of the target mRNA 3′-UTR, and on the functional relevance of each target gene in each cell type. This might be totally different at different stages of the myeloid lineage or in different AML subtypes. Finally, the studies of Song et al. primarily employed overexpression experiments, which can potentially lead to effects quite different to those observed at physiologic levels, while the work by Shen et al. included both gain-of-function and loss-of function model systems.

Thus, is there a limitation of the model system or rather the species? Jiang et al. provided strong evidence of a tumor-suppressive function of miR-22 in various leukemic mouse models, whereas enforced expression of miR-22 leads to a delayed leukemia onset and a longer survival. Looking at the biology of leukemic transformation events, it is often a matter of being in the right place at the right time. An example is the myeloid transcription factor CEBPA: while under normal conditions it functions as a typical tumor suppressor and master regulator of myelopoiesis [13], it has been reported that its expression is crucial for mixed lineage leukemia (MLL) rearrangements to induce leukemia in mice [14, 15]. Without a differentiation stimulus, the leukemia-initiating cells fail to develop into malignant blasts and cannot induce leukemia. In contrast to this, a knockout of the CEBPA gene in nonleukemic cells results in a block of granulocytic differentiation and an accumulation of blasts in the bone marrow [16]. That could also be the case for miR-22.

**Fig 1:**
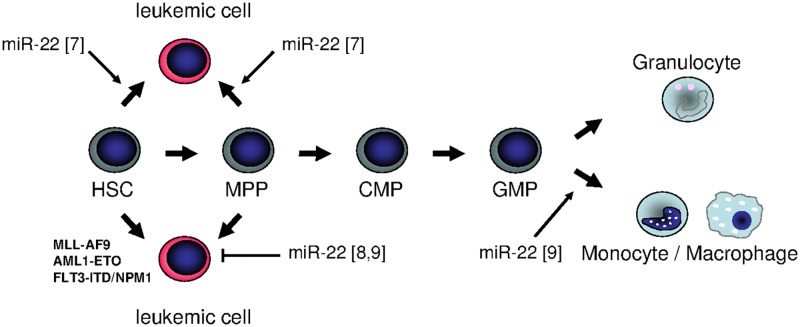
Summary of different functions of miR-22 in hematopoiesis: is miR-22 an oncogenic tumor suppressor or rather a tumor-suppressive oncogene? In September 2016’s issue of *PLOS Genetics*, Shen et al. revealed the potential of miR-22 to trigger monocytic differentiation in healthy and leukemic cells [9]. These data are supported by the finding by Jiang et al., who demonstrated that enforced miR-22 expression is sufficient to delay disease onset in different mouse models for acute myeloid leukemia [8]. In contrast, it was previously reported that miR-22 was up-regulated in myeloid disease, and that overexpression of miR-22 in normal stem and progenitor cells led to the development of a myeloid leukemia–like phenotype [7].

In summary, miR-22 seems to show a Janus-faced nature in hematopoiesis: it can be both oncogenic and tumor-suppressive, depending on the specific individual background. In fact, further studies are obligatory to examine the function of miR-22 in different backgrounds within the myeloid lineage. It might be that its role in early stem cells differs from that in committed myeloid progenitors, and that a combination with classical leukemia–associated genomic alterations results in a totally different phenotype (Fig 1). These open questions clearly illustrate that nature is not always black and white, and sometimes an additional view behind the horizon is necessary to elicit all her secrets.
