# Correction: The Janus-faced Nature of miR-22 in Hematopoiesis: Is It an Oncogenic Tumor Suppressor or Rather a Tumor-Suppressive Oncogene?

**DOI:** 10.1371/journal.pgen.1012281

**Published:** 2026-08-25

**Authors:** The PLOS Genetics Editors

This Perspective [1] discusses research findings presented in a *PLOS Genetics* Research Article [2] that has since been retracted [3]. Statements in [1] supported only by the retracted article [2, 3] should be disregarded.

The *PLOS Genetics* Editors consider that [1] continues to provide a useful perspective on the role of miR-22 in hematopoiesis after the findings in [2] are dismissed.
